# Investigating Factors Associated with Thymic Regeneration after Chemotherapy in Patients with Lymphoma

**DOI:** 10.3389/fimmu.2016.00654

**Published:** 2016-12-27

**Authors:** Dao-Ping Sun, Li Wang, Chong-Yang Ding, Jin-Hua Liang, Hua-Yuan Zhu, Yu-Jie Wu, Lei Fan, Jian-Yong Li, Wei Xu

**Affiliations:** ^1^Department of Hematology, The First Affiliated Hospital of Nanjing Medical University, Jiangsu Province Hospital, Collaborative Innovation Center for Cancer Personalized Medicine, Nanjing Medical University, Nanjing, China; ^2^Department of Hematology, Jining No.1 People’s Hospital, Jining, China; ^3^Department of Nuclear Medicine, The First Affiliated Hospital of Nanjing Medical University, Jiangsu Province Hospital, Nanjing, China

**Keywords:** thymus, hyperplasia, regeneration, lymphoma, chemotherapy, interleukin-7 receptor-α, single-nucleotide polymorphisms

## Abstract

The factors involved in thymus regeneration after chemotherapy has not been sufficiently explored. This study was aimed to identify the clinical characteristics and single-nucleotide polymorphisms in the gene (*IL7R*) encoding IL-7Rα associated with thymus renewal after chemotherapy in Chinese Han individuals with lymphoma. The dynamics of thymic activity in 134 adults with Hodgkin lymphoma (HL) and B cell lymphoma from baseline to 12 months post-chemotherapy were analyzed by assessing thymic structural changes using serial computed tomography scans and correlating these with measurements of thymic output by concurrent analysis of single-joint T-cell receptor excision circles (sjTREC) and CD31^+^ recent thymic emigrants (RTE) in peripheral blood. The association of clinical variables and *IL7R* polymorphisms with the occurrence of rebound thymic hyperplasia (TH) and the recovery of thymic output following chemotherapy were evaluated. Thymic regeneration was observed, with the evidence that TH occurred in 38/134 (28.4%) cases, and thymic output, assessed by CD31^+^ RTE numbers and sjTREC content, recovered to baseline levels within 1 year after the end of therapy. The frequencies of the T allele and TT + GT genotype of rs7718919 located in the promoter of *IL7R* were significantly higher in patients with TH compared with those without TH (*P* = 0.031 and 0.027, respectively). In contrast, no significant difference was found between two groups with respect to the distribution of allele and genotype frequencies of rs6897932. By general linear models repeated-measure analysis, rs7718919 and rs6897932 were determined to exert no significant effects on the recovery of thymic output after therapy. Univariate analysis revealed host age under 30, the diagnosis of HL, baseline thymic index and CD31^+^ RTE counts, and rs7718919 genotype as potential predictors for TH after chemotherapy (*P* < 0.05); after multivariate adjustment, only host age was independently associated with the occurrence of TH (odds ratios = 4.710, 95% confidence intervals: 1.727–12.845, *P* = 0.002). These findings indicate that patient age is an independent predictor for thymic regrowth after chemotherapy, which should promote awareness among physicians to make a timely diagnosis of TH in young adults and help physicians to prioritize intervention strategies for thymus rejuvenation in this population.

## Introduction

Atrophy of the thymus caused by cytotoxic drugs and glucocorticoid hormones remains a primary obstacle to full immune recovery following chemotherapy, which is dependent on high thymic output of new recent thymic emigrants (RTE) to replenish the naïve T cell pool (1, 2). Failure of restoration of thymus function may lead to increased risks of infections and tumor recurrence, particularly in elderly patients where the thymus is atrophied (1, 3). Enlargement of the thymus above baseline following chemotherapy, known as rebound thymic hyperplasia (TH), sometimes occurs during recovery from chemotherapy (4). TH may be related with robust thymic regeneration and is characterized by an increase in thymic size and density, concurrent with the restoration of thymic output of T cells (5–7). This phenomenon is common in children and adolescents and can occasionally be observed in young adults; however, it is rare in older patients (4, 8–11). Therefore, more effort should be made toward restoring thymic function in post-pubertal patients. However, the factors involved in thymus atrophy and regeneration are not fully understood, and approaches to stimulate rejuvenation of the thymus remain limited (12).

Clinical factors associated with thymic regrowth after chemotherapy have been explored in previous studies. Information on the occurrence of TH following chemotherapy obtained from different age groups revealed that the renewal ability of the thymus may be influenced by host age, and TH after chemotherapy is more common in younger populations with greater amounts of residual thymic tissues and higher thymic activity (4–11). Whereas, thymic regrowth is not likely associated with the tumor types and treatment. It can occur following treatment for various malignancies, of which malignant lymphoma is the most common (8). Besides, TH after cessation of chemotherapy does not appear to be influenced by the degree of lymphocyte depletion, but appears to be a common response to the withdrawal of chemotherapy (4, 6). There is also evidence that TH after chemotherapy maybe associated with endocrine dysfunctions (13). However, the factors mentioned above need to be further validated, since the knowledge of TH after chemotherapy was mainly obtained from analysis of small patient groups, and other potential factors related with TH have not been elicited.

Interleukin-7 (IL-7) acts as a non-redundant cytokine in thymic development (14). Under conditions of lymphopenia, it could optimize the long-term recovery of T cell diversity by restoring thymic function (15, 16). In patients with human immunodeficiency virus (HIV) infection, those with higher baseline levels of IL-7 had a higher incidence of TH after therapy (17). Patients with rheumatoid arthritis and type 1 diabetes demonstrating positive responses to treatment exhibited raised plasma IL-7 levels concurrent with renewed thymopoeisis (18, 19). Thus, IL-7 may be an important factor in thymic regeneration after injury. The responsiveness of IL-7 is dependent on the expression of the IL-7 receptor (IL-7R), which is a heterodimer consisting of the common γ-chain and the α-chain (IL-7Rα) (20, 21). The gene encoding IL-7Rα (*IL7R*) is polymorphic, and single-nucleotide polymorphisms (SNPs) in *IL7R* could potentially affect its transcription, thereby influencing IL-7Rα expression levels and signal transduction (22–26). *IL7R* polymorphisms are likely to modulate the regulation, differentiation, and function of T cell subsets and are associated with the susceptibility to autoimmune diseases, the pathogenesis of graft-versus-host disease after hematopoietic stem cell transplantation (HSCT), and T cell repopulation after lymphocytopenia caused by HIV infection and HSCT (22–32). Moreover, SNPs in *IL7R* may influence thymic T cell development in patients with multiple sclerosis (MS) (25), indicating a possible role for these SNPs in the process of thymic regeneration after chemotherapy.

Considering these elements, the present study was aimed at examining clinical predictors for the occurrence of TH in a group of adult patients undergoing chemotherapy for lymphoma and exploring the possible contribution of *IL7R* polymorphisms to thymic renewal capacity by detecting possible links between *IL7R* SNPs and the recovery of thymic volume and output function after chemotherapy.

## Materials and Methods

### Patients

Chinese Han patients with Hodgkin lymphoma (HL) and B cell lymphoma (*n* = 193) admitted to the Department of Hematology, the First Affiliated Hospital of Nanjing Medical University between January 2012 and June 2015 were included in this study. All subjects provided written informed consent in accordance with the requirements of the Declaration of Helsinki. The study was approved by the Ethics Committee of the First Affiliated Hospital of Nanjing Medical University. All patients accepted chemotherapy for lymphoma at diagnosis. For patients with HL, ABVD (adriamycin, bleomycin, vinblastine, and dacarbazine) regimen was administered. Patients with diffuse large B cell lymphoma (DLBCL) were treated with R-CHOP (rituximab, cyclophosphamide, doxorubicin, vincristine, and prednisone) or R-DA-EPOCH (rituximab, etoposide, prednisone, vincristine, cyclophosphamide, and doxorubicin) regimen. Patients with follicular lymphoma (FL) or marginal zone lymphoma (MZL) accepted R-CHOP regimen mentioned above or R-COP (rituximab, cyclophosphamide, vincristine, and prednisone) regimen. Patients with Burkitt lymphoma (BL) received R-hyperCVAD (rituximab, cyclophosphamide, vincristine, doxorubicin, and dexamethasone) therapy alternating with R-MA (rituximab, methotrexate, and cytarabine). CT examinations were performed before (baseline), during (after three cycles of chemotherapy), and after (0, 3, 6, 9, and 12 months post-chemotherapy) treatment. Simultaneously, blood samples were collected for flow cytometric analyses and peripheral blood mononuclear cells (PBMCs) separated by density gradient centrifugation. Patients (*n* = 28) who had no response to therapy, or had disease progression within 1 year after the end of treatment, were excluded. Patients (*n* = 15) who responded to chemotherapy and underwent HSCT as first-line consolidation were also excluded. For 16 patients, it was not possible to determine thymus size because of the growth of the lymphoma. The remaining 134 patients (82 DLBCL, 15 HL, 14 FL, 10 MZL, 4 BL, and 9 others), aged 18−67 (median 41) years, were included.

### Thymus Imaging

Serial analyses of structural changes of the thymus were performed by reviewing CT images. Thymic size was scored using a thymic index on a scale from 0 to 5 as described elsewhere (6): 0, no soft tissue; 1, minimal soft tissue, barely recognizable; 2, minimal soft tissue, more obvious; 3, moderate soft tissue; 4, moderate soft tissue of greater extent, almost mass-like; and 5, mass-like appearance, suggesting hyperplasia or thymoma. Thymic enlargement, or subsequent regression, was defined as a change in score of at least 1 on this 0–5 scale. Enlargement of the thymus over baseline in the absence of any clinical, laboratory, or radiological signs of disease progression was interpreted as TH.

### CD31^+^ RTE Detection

For immunophenotypic analysis, whole blood samples were stained by four-color technique using anti-CD45RA-FITC, anti-CD31-PE, anti-CD4-PerCP, and anti-CD3-APC monoclonal antibodies and appropriate isotope controls (Becton Dickinson, USA). Erythrocytes were lysed with lysis solution (Becton Dickinson, USA). Lymphocyte data were analyzed by flow cytometry using a FACSCalibur (Becton Dickinson, USA). After gating for CD3^+^ and CD4^+^ cells, the percentage of CD31^+^ cells among all CD45RA^+^CD4^+^ T cells was analyzed with CELLQuestTM software (Becton Dickinson, USA) as previously described (33). Whole blood lymphocyte counts were performed with an automated analyzer, and the absolute numbers of CD31^+^ RTEs were determined by multiplying the total lymphocyte count by the percentage of CD31^+^CD45RA^+^CD4^+^ T cells.

### *IL7R* Genotyping

Genomic DNA samples were extracted from PBMCs using a QIAamp DNA Blood Midi Kit (Qiagen, Germany), according to the manufacturer’s instructions. On the basis of a literature search, four SNPs were chosen as our primary targets of investigation, including rs6897932 in exon 6, and rs7718919, rs11567685, and rs11567686 in the promoter region, of *IL7R* (22–25). Genotyping was performed by DNA sequencing. Briefly, the amplicons containing the promoter and exon 6 regions of *IL7R* were PCR-amplified from genomic DNA samples using primer sequences previously reported (22). PCR products were then purified by polyethylene glycol precipitation. Next, DNA sequencing was performed in both directions using the ABI Prism Big Dye Terminator version 3.1 sequencing kit and an ABI 3730XL Genetic Analyzer. Sequencing results were analyzed using Chromas 2.22 software (Technelysium, Australia).

### Single-Joint T-Cell Receptor Excision Circles (sjTREC) Analysis

Serial quantification of sjTREC in the DNA of PBMCs was performed using a TaqMan real-time quantitative PCR assay and a StepOnePlus instrument (Applied Biosystems, USA), as previously described (5). A standard curve based on a plasmid preparation containing the sjTREC target sequence was plotted, and sjTREC values for samples were calculated using StepOne software (Applied Biosystems, USA). Samples were analyzed in triplicate, and median values calculated. Data are expressed as TRECs/10^6^ cells.

### Statistics

Continuous variables are expressed as means ± SD and categorical variables as number of cases (percentage). Independent *t* tests or Mann–Whitney *U* tests were used to evaluate differences in numerical data. Chi-square or *Fisher’s* exact tests were used to assess differences in categorical data and to compare genotype and allele frequencies between patients with and without TH. Odds ratios (OR) and 95% confidence intervals (CI) were calculated for the assessment of risk factors. Genotyping data were analyzed for Hardy–Weinberg equilibrium (HWE) and linkage disequilibrium (LD) using HaploView 4.2. LD blocks were identified using the CI setting. Univariate and multivariate logistic regression models were performed to investigate the associated factors for TH after chemotherapy. Variables with *P* value < 0.10 in the univariate analysis were selected for the multivariate analysis. The changes of thymic output at different time points were assessed by general linear models repeated-measure analysis using the statistical test within-subject contrasts. The effect of *IL7R* SNPs on thymic output recovery was evaluated by general linear models repeated-measure analysis using between-subject contrasts. Data analysis was performed using SPSS21 statistical software. Values of *P* < 0.05 were considered to be significant.

## Results

### The Occurrence of TH after Chemotherapy

Thymic hyperplasia was observed in 38/134 (28.4%) patients within intervals ranging from 1 to 10 months (median, 4 months) after the cessation of chemotherapy. Compared with those without TH (*n* = 96), patients with TH (*n* = 38) after chemotherapy were much younger, with larger thymic volumes, and higher thymic output before treatment (*P* < 0.05). Moreover, patients with TH were more commonly diagnosed with HL treated with chemotherapy only (*P* < 0.05). However, no significant differences were observed in sex, disease stage, and the pool size of peripheral CD4^+^ T-cells at baseline between two groups (*P* > 0.05). The clinical characteristics of these patients are presented in Table 1.

**Table 1:** **Baseline characteristics of patients with and without thymic hyperplasia (TH) after chemotherapy**.

Characteristics	Patients with TH (*n* = 38)	Patients without TH (*n* = 96)	*P*-value
Age (years), median (IOR)	30 (18–53)	48 (18–67)	<0.001
Gender, *n* (%)			
Female	20/38 (53)	59/96 (61)	0.436
Male	18/38 (47)	37/96 (39)	
Disease type, *n* (%)			
DLBCL	18/38 (47)	64/96 (67)	0.038
HL	8/38 (21)	7/96 (8)	
Others	12/38 (32)	25/96 (26)	
Disease stage, *n* (%)			
I–II	18/38 (47)	33/96 (34)	0.163
III–IV	20/38 (53)	63/96 (66)	
Treatment, *n* (%)			
Chemotherapy	8/38 (21)	7/96 (8)	0.023
Chemotherapy + rituximab	30/38 (79)	89/96 (92)	
CD4^+^ T cells counts (×10^9^/L)	642.27 ± 385.79	577.67 ± 365.14	0.756
Thymic index, *n* (%)			
0	3/38 (8)	45/96 (47)	<0.001
1	10/38 (26)	23/96 (24)	
2	19/38 (50)	21/96 (22)	
3	5/38 (13)	6/96 (6)	
4	1/38 (3)	1/96 (1)	
Thymic output			
CD31^+^RTE (×10^9^/L)	194.85 ± 158.31	98.12 ± 84.87	0.036
sjTREC (copies/10^6^ PBMCs)	10,381.09 ± 8,393.22	5,109.70 ± 7,162.70	0.042

*DLBCL, diffuse large B cell lymphoma; HL, Hodgkin lymphoma; RTE, recent T cell emigrant; sjTREC, single-joint T-cell receptor rearrangement excision circle; PBMCs, peripheral blood mononuclear cells*.

### Recovery of Thymic Output after Chemotherapy

**Figure 1:**
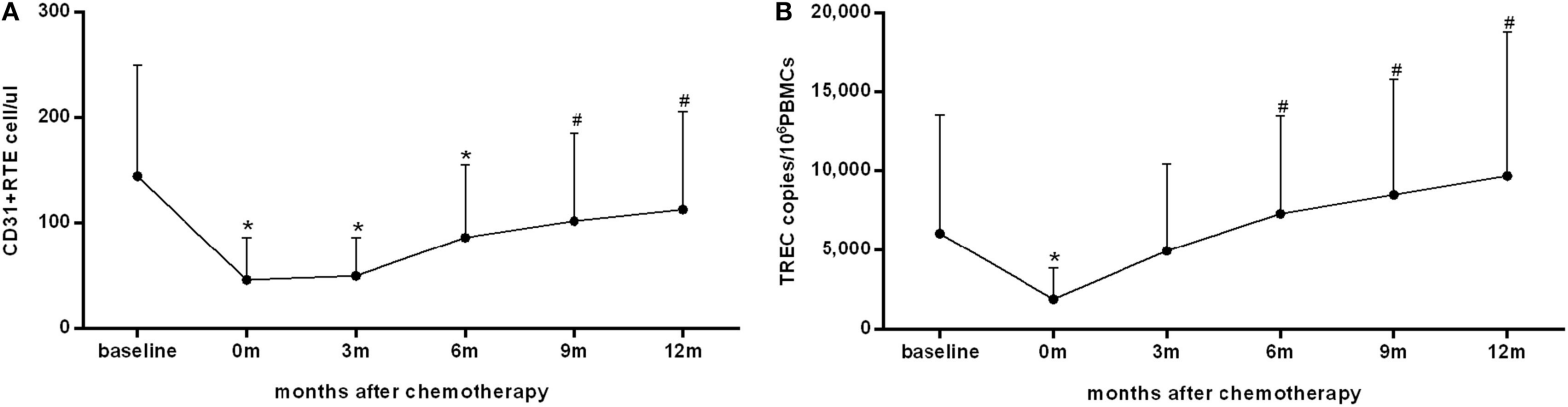
**The recovery of thymic output measured by CD31^+^ recent thymic emigrants (RTE) (A) and single-joint T-cell receptor excision circles (sjTREC) (B) in peripheral blood within 1 year after the end of chemotherapy in 84 patients**. Both the CD31^+^ RTE counts and sjTREC levels decreased to the nadir at the end of treatment and recovered within 1 year of follow-up. Data are shown as means ± SD. *P* values were assessed by general linear model analysis for repeated-measure data. *P < 0.001 vs. baseline levels; ^#^*P* < 0.05 vs. the nadir at the end of treatment.

The change in thymic output during and after chemotherapy was analyzed in 84 patients with available CD31^+^ RTE and sjTREC data at all follow-up time points (Figure 1). Consistent with our previous findings (5), both the numbers of CD31^+^ RTE and levels of sjTREC approached a nadir at the end of treatment (*P* < 0.001). During follow-up, CD31^+^ RTE counts and sjTREC levels rose significantly and reached pretreatment levels at 9 and 6 months after the end of chemotherapy, respectively (*P* = 0.035 and 0.001, respectively).

### The Effect of *IL7R* Polymorphisms on TH after Chemotherapy

Genotypes for rs11567686 did not conform to HWE (*P* = 0.029) and were excluded from further analyses. Genotypes of the three remaining SNPs were all consistent with HWE (*P* > 0.05); however, the minor allele frequency of rs11567685 (0.019%) was too low for differences between the two groups to be meaningfully analyzed. Therefore, allele and genotype frequencies for rs7718919 and rs6897932 in patients with and without TH were compared (Table 2).

**Table 2:** **Genotype distribution and allele carriage rates of two single-nucleotide polymorphisms (SNPs) in the gene encoding IL-7Rα in patients with and without thymic hyperplasia (TH) following chemotherapy**.

SNP locus	Genotype/allele	Patients with TH (*n* = 38)	Patients without TH (*n* = 96)	Odds ratios [95% confidence intervals]	*P*-value
rs7718919	GG GT TT	24 (0.632) 12 (0.316) 2 (0.053)	78 (0.812) 15 (0.156) 3 (0.031)		0.084
	TT GG + GT	2 (0.053) 36 (0.947)	3 (0.031) 93 (0.969)	1.722 [0.276–10.737]	0.622
	GT + TT GG	14 (0.368) 24 (0.632)	18 (0.187) 78 (0.813)	2.528 [1.097–5.826]	0.027
	T G	16 (0.211) 60 (0.789)	21 (0.109) 171 (0.891)	2.171 [1.064–4.433]	0.031
rs6897932	CC CT TT	27 (0.711) 11 (0.289) 0 (0.000)	59 (0.615) 34 (0.354) 3 (0.031)		0.383
	CC + CT TT	38 (1.000) 0 (0.000)	93 (0.969) 3 (0.031)	1.413 [1.265–1.578]	0.577
	CC CT + TT	27 (0.711) 11 (0.289)	59 (0.615) 37 (0.385)	1.539 [0.683–3.469]	0.325
	C T	65 (0.855) 11 (0.145)	152 (0.792) 40 (0.208)	1.555 [0.751–3.220]	0.282

The minor T allele of rs7718919 was significantly more frequent in patients with TH compared with those without TH (*P* = 0.031, OR = 2.171, 95% CI 1.064–4.433, T vs. G). We also detected a significant difference under a recessive model with regard to the distribution of rs7718919 genotype frequencies between patients with and without TH (*P* = 0.027, OR = 2.528, 95% CI 1.097–5.826, TT + GT vs. GG); however, no significant evidence was detected under a dominant model (*P* = 0.622, OR = 1.722, 95% CI 0.276–10.737, TT vs. GG + GT). Moreover, no significant difference was found with respect to the distribution of allele and genotype frequencies of rs6897932 between subjects with and without TH (*P* = 0.383 and 0.282, respectively).

Due to high diversity and weak LD (*r*^2^ = 0.365) between rs7718919 and rs6897932, haplotype analyses were not possible in our study cohort.

### The Effect of *IL7R* Polymorphisms on the Recovery of Thymic Output after Chemotherapy

As previously shown in Ref. (4), thymic regeneration after chemotherapy manifests as an increase in thymic volume, concurrent with the restoration of thymopoiesis. We investigated the influence of rs7718919 and rs6897932 on the renewal of thymopoiesis following chemotherapy in 84 patients with thymic output data available for all follow-up time points.

The effect of rs7718919 genotypes was tested using a recessive model (TT + GT vs. GG), due to few cases carrying the minor allele T. By general linear models repeated-measure analysis, no impact of rs7718919 genotypes was found on the recovery of CD31^+^ RTEs counts and sjTREC levels within 1 year of follow-up (*P* = 0.743 and 0.642, respectively) (Figure S1 in Supplementary Material). Similarly, the effect of rs6897932 genotype was tested using a recessive model (CC vs. CT + TT) and no impact was found on the restoration of thymic output measured by both CD31^+^ RTEs counts and sjTREC levels (*P* = 0.913 and 0.896, respectively) (Figure S2 in Supplementary Material). Baseline characteristics, including age, sex, disease type and stage, CD4^+^ cell counts, and output function at baseline were comparable between the two groups with different genotypes (*P* > 0.05) (Table S1 in Supplementary Material).

### Analysis of Factors Associated with TH after Chemotherapy

The univariate analysis results showed significant differences between patients with and without TH in terms of host age, disease type, pretreatment thymic size and CD31^+^ RTE counts, and rs7718919 genotype (*P* < 0.05) (Table 3). Then, by multivariate analysis, it was concluded that host age under 30 is the only independent predictive factor for the occurrence of TH after chemotherapy (*P* = 0.002, OR = 4.710, 95% CI: 1.727–12.845). A higher probability of thymic regrowth following chemotherapy was observed in subjects with the T allele of rs7718919; however, this trend did not reach statistical significance (*P* = 0.093, OR = 2.308, 95%CI: 0.875–6.091) (Table 3).

**Table 3:** **The univariate and multivariate analysis of factors influencing the occurrence of thymic hyperplasia after chemotherapy**.

Parameters	Univariate analysisOdds ratios (OR) [95% confidence intervals (CI)]	*P*-value	Multivariate analysisOR [95% CI]	*P*-value
Age ≤30 years	8.054 [3.415–18.991]	<0.001	4.710 [1.727–12.845]	0.002
Gender: female	1.435 [0.673–3.062]	0.350		
Disease type: HL	3.390 [1.134–10.140]	0.029	2.279 [0.646–8.036]	0.200
Disease stage: I−II	1.718 [0.801–3.687]	0.165		
Baseline thymic index ≥2	4.670 [2.059–10.412]	<0.001	2.087 [0.774–5.628]	0.146
Baseline CD4^+^ T cell counts ≥600 × 10^9^/L	1.950 [0.445–8.548]	0.376		
Baseline CD31^+^ RTE counts ≥150 × 10^9^/L	3.896 [1.059–14.326]	0.041	2.160 [0.430–10.845]	0.350
Baseline sjTREC levels ≥7,000 copies/10^6^ PBMCs	4.050 [0.974–16.842]	0.054	3.052 [0.277–18.040]	0.362
Rs7718919 genotype: TT + GT	2.528 [1.097–5.826]	0.029	2.308 [0.875–6.091]	0.093

*HL, Hodgkin lymphoma; RTE, recent T cell emigrant; sjTREC, single-joint T-cell receptor rearrangement excision circle; PBMCs, peripheral blood mononuclear cells*.

## Discussion

Reactive TH after chemotherapy is a well-documented phenomenon in children, adolescents, and young adults, but is rare in older patients, suggesting a high thymic regenerative capacity in the young (4, 5, 8–11). However, by monitoring thymic output, it was determined that substantial output is maintained into late adulthood despite the decline of thymic function with age, and even the aged thymus retains the ability to renew thymopoiesis (34). This study further explored the influence of age on the renewal capacity of the thymus following chemotherapy. In the cohort of adults accepting chemotherapy for lymphoma, TH was observed in 38 (28.4%) cases aged 18–53 years (median 30), who were much younger, with larger thymic volumes and higher thymic output before treatment than those without TH, suggesting a high renewal capacity was retained in the thymus of young adults. In contrast, TH was observed in none of the elderly, possibly due to a decreased regenerative capacity of the thymus with age, which could represent a decline in the intrinsic capacity of lymphopoietic stem cells and/or changes in the thymic microenvironment (7). Besides, by multivariate analysis, patient age under 30 was revealed as an independent predictor for the occurrence of TH, further supporting the conclusion that thymic renewal capacity after chemotherapy is age-dependent. Consistent with this, we have revealed in an earlier study that, the majority (70%) of TH following chemotherapy was found in individuals under 30 years, although it can occasionally be observed in middle-aged patients (10). Furthermore, the renewal of thymopoiesis following chemotherapy was evaluated in the present study. The fast restoration of thymic output within 1 year of follow-up observed in these adults suggested a renewed thymopoiesis. Thymus regeneration, manifesting as partial reversal of thymic atrophy concurrent with a renewed thymopoiesis, in young adults undergoing chemotherapy would contribute to immune recovery, enhancing the clinical relevance of developing strategies to booster thymic activity in this population (2, 5, 12).

Clinical features except for patient age associated with thymic regrowth after chemotherapy have not been studied in-depth. Based on previous observations, it was seemed that, TH is neither tumor nor treatment specific (4, 6, 8). This study revealed that, patients with TH after chemotherapy were more commonly diagnosed with HL than those without TH. While, by multivariate analysis, the diagnosis of HL was not associated with the occurrence of TH independently, it can be conceived that, the lower age of patients with HL may contribute to a higher probability of thymic regrowth. Whether thymic regrowth following chemotherapy is correlated with the biological properties of the tumors remains unknown. It should be reminded that, the influence of rituximab usage on the occurrence of TH post-chemotherapy cannot be excluded, since HL patients were treated with chemotherapy only, in contrast with patients with B cell lymphoma accepted chemotherapy combined with rituximab. Emerging data suggest that rituximab has an inhibitory effect on effector T cells and could induce regulatory T cells expansion, which may be related to B cell deprivation resulting in impaired antigen-presenting cells activity and reduced production of co-stimulatory and immuno-modulatory molecules taking part in T cell activation and interactions; besides, it could exert a direct effect on T cells by binding to a specific T cell subset expressing CD20 (35). A recent study confirmed that, CD20 was also expressed on mature medullary thymocytes (36). Therefore, rituximab may intervene T cell development in the thymus by targeting CD20^+^ thymocytes. Unfortunately, this study design precluded an analysis for the effect of rituximab. Whether the use of rituximab could affect thymic activity and thus delay the regeneration of the thymus in patients with B cell lymphoma deserves further studies. Moreover, TH was demonstrated to be caused by chemotherapy-induced gonadal atrophy, which results in increased luteinizing hormone secretion (13), and sex steroid ablation has been regarded as an important candidate strategy for the improvement of thymic regeneration (12). Thus, the sex-related endocrine status is presumed to influence the renewal capacity of the thymus after chemotherapy. However, sex ratio was comparable in cases with and without TH in this study, excluding sex as a predictor for TH. Measurement of sex-related endocrine function is warranted to clarify this issue. In addition, the occurrence of TH in this cohort of adults we studied was not influenced by the size of peripheral CD4^+^ T cell pool as expected, since renewed thymopoiesis correlated with TH play a limited role in the early repopulation of peripheral CD4^+^ T cells following chemotherapy in adults (10).

The mechanisms for the reconstitution of thymic cellularity, accompanied by the restoration of thymic T cell development, after the removal of chemo-therapeutic toxicity is complex (37, 38). Interleukin-7, a growth and anti-apoptotic factor for T cells, was found to be associated with thymus enlargement in HIV patients undergoing antiretroviral therapy, suggesting an important role of IL-7 in thymus regrowth (17). Consistent with this findings, administration of recombinant human IL-7 was shown to increase the numbers of RTEs and levels of TRECs and results in a broadening of the T-cell receptor repertoire, indicating increased thymopoiesis (39). The response of thymocytes to IL-7 correlates with IL-7Rα expression. Accordingly, this study focused on four specific SNPs within the *IL7R* locus, known to influence the IL-7Rα expression on T cells (23–25), and explored their potential contributions to the thymic regeneration after chemotherapy in adults with lymphoma. It was found that the frequencies of the minor allele T and the TT + GT genotype of rs7718919, located in the promoter region of *IL7R*, were higher in patients with TH compared with those without TH. These results provided a possible link between rs7718919 and thymus enlargement after chemotherapy. Considering rs7718919 could potentially influence the expression of IL-7Rα as a transcription factor binding site (30), it is conceivable that it may affect IL-7-mediated regulation of thymocytes, and thus be implicated in thymic regrowth. However, this association between rs7718919 and the occurrence of TH was not confirmed by multivariate analysis. Moreover, we did not observe an accelerated recovery of thymic output after chemotherapy in patients with TT + GT genotype compared with GG genotype carriers, suggesting that the presence of the rs7718919 T allele had limited effects on the restoration of thymopoiesis. Consistent with these results, Shamim et al. (31) were unable to demonstrate any association between *IL7R* polymorphisms and TREC levels before or after HSCT in a Danish cohort. Nevertheless, the influence of rs7718919 polymorphisms on the renewal of thymopoiesis should be carefully evaluated. As IL-7Rα expression is finely tuned and differentially regulated during thymocyte development (21), it is important to better understand which thymocyte subset could be influenced by the altered IL-7 signaling associated with rs7718919 and to what extent this could affect thymic T cell development.

This study also investigated rs6897932, a missense polymorphism located in exon 6 of *IL7R*, which encodes the trans-membrane region of IL-7Rα. The C allele of rs6897932 is associated with increased skipping of exon 6, resulting in a higher ratio of membrane-bound vs. soluble receptor, which could cause reduced IL-7Rα expression on T cells (26). In a Danish cohort, homozygosity for the rs6897932 T-allele was associated with more rapid CD4^+^ T-cell recovery after the initiation of antiretroviral therapy (28). Unfortunately, the design of that study did not allow evaluation of whether the increase in CD4^+^ T cells was caused by increased thymopoiesis or expansion of the peripheral T-cell pool. Our study revealed no significant association of rs6897932 polymorphisms with either thymic enlargement or the restoration of thymic output, indicating a limited role for rs6897932 variants in thymopoiesis recovery after chemotherapy. This is consistent with the report by Broux et al. (25) of no influence of rs6897932 polymorphisms on thymopoiesis, as measured by the frequency of CD31^+^ RTEs in MS patients and healthy controls. Further studies investigating the potential effects of rs6897932 on the expression of IL-7Rα and its possible influence on the affinity and/or signaling of IL-7Rα in various thymocyte subsets are warranted to elucidate its role on thymopoietic activity. In addition, rs11567685, located in the promoter region of *IL7R*, was included in this study. It could potentially influence thymic development and peripheral homeostasis of T cells by altering the expression of IL-7Rα on CD4^+^ T cells in MS patients (25). Unfortunately, the effect of rs11567685 on the recovery of thymic cellularity and function after chemotherapy in this study cannot be evaluated, due to its low minor allele frequency.

This study has some limitations. Our observations were based on relatively few cases, and this may have masked significant effects due to lack of statistical power. In addition, thymic output measured by determination of sjTREC and CD31^+^ RTE may be influenced by the proliferation of peripheral T cells, potentially leading to an underestimation of thymic activity (40, 41). Hence, future studies with larger sample sizes and more accurate tools for thymic output measurement are necessary to further define the factors associated with thymic regrowth after chemotherapy. Moreover, evaluating the effect of rituximab usage on thymus regeneration following chemotherapy by comparing the occurrence of TH and the recovery of thymic output in two groups with comparable clinical features, including age, sex, disease type and stage, thymic size, and output function at baseline, is warranted in future studies.

To conclude, these findings suggest that patient age is an independent predictor for thymic regrowth after chemotherapy, which should promote awareness among physicians to make a timely diagnosis of TH in young adults and help physicians to prioritize intervention strategies for thymus rejuvenation after chemotherapy in this population. Besides, the potential link between the *IL7R* SNP and thymic regrowth after chemotherapy warrant further investigations, given the key roles of IL-7 and IL-7Rα in thymic development. These results will contribute to an improved understanding of the mechanisms involved in thymic regeneration.

## Ethics Statement

Ethics approval and consent to participate was obtained. In accordance with the 1975 guidelines of the Declaration of Helsinki and after obtaining approval for the study from the Ethics Committee of the First Affiliated Hospital of Nanjing Medical University, the medical staff of each participating center informed their patients of the potential risks and benefits of the planned treatment and the need for periodical clinical and laboratory checkups in detail. Informed consent was obtained from all the subjects involved in this study.

## Author Contributions

WX and J-YL conceived and designed the study; D-PS, LW, C-YD, and J-HL contributed to the experimental design and provided intellectual input; D-PS and Y-JW performed experiments; LW, H-YZ, and LF contributed patients and provided clinical data; D-PS and LW analyzed data and wrote the manuscript.

## Conflict of Interest Statement

The authors declare that the research was conducted in the absence of any commercial or financial relationships that could be construed as a potential conflict of interest.
