# Supplementary material for: Investigating Factors Associated with Thymic Regeneration after Chemotherapy in Patients with Lymphoma
Source: Front Immunol. 2016 Dec 27;7:654. doi: 10.3389/fimmu.2016.00654 (PMC5186774; doi:10.3389/fimmu.2016.00654)
Supplement: Supplementary file 1 [file Table_1.DOCX]

**Investigating factors associated with thymic regeneration after chemotherapy in patients with lymphoma**

Dao-Ping Sun^1,3^*, Li Wang^1^*, Chong-Yang Ding^2^, Jin-Hua Liang^1^, Hua-Yuan Zhu^1^, Yu-Jie Wu^1^, Lei Fan^1^, Jian-Yong Li^1^ and Wei Xu^1^

*Dao-Ping Sun and Li Wang contributed equally to this work.

^1^Department of Hematology, the First Affiliated Hospital of Nanjing Medical University, Jiangsu Province Hospital, Collaborative Innovation Center for Cancer Personalized Medicine, Nanjing Medical University, Nanjing, China;

^2^Department of Nuclear Medicine, the First Affiliated Hospital of Nanjing Medical University, Jiangsu Province Hospital, Nanjing, China

^3^Department of Hematology, Jining No.1 People’s Hospital, Jining, China;

Correspondence author: Dr Wei Xu, Department of Hematology, the First Affiliated Hospital of Nanjing Medical University, Jiangsu Province Hospital, Nanjing 210029, China.

Telephone: +86-25-83781120; Fax: +86-25-83781120**.**

E-mail: [xuwei10000@hotmail.com](mailto:xuwei10000@hotmail.com)

**Figure legends**

**Figure S1.** The influence of rs7718919 genotypes on the recovery of thymic output following chemotherapy. CD31^+^ recent thymic emigrants (RTE) **(A)** counts and single-joint T-cell receptor excision circles (sjTREC) **(B)** levels in the peripheral blood were measured serially before (baseline) and after (0, 3, 6, 9, and 12 months post-chemotherapy) treatment in 21 patients with GT + TT genotype (●) and 63 patients with GG genotype (■). By general linear models repeated measure analysis for repeated measure data, rs7718919 was determined to exert no significant effects on the recovery of CD31^+^ RTEs counts and sjTREC levels after the cessation of chemotherapy (*p* > 0.05). Data are shown as means ± SD.

**Figure S2.** The influence of rs6897932 genotypes on the recovery of thymic output following chemotherapy. CD31^+^ recent thymic emigrants (RTE) **(A)** counts and single-joint T-cell receptor excision circles (sjTREC) **(B)** levels in the peripheral blood were measured serially before (baseline) and after (0, 3, 6, 9, and 12 months post-chemotherapy) treatment in 29 patients with CT + TT genotype (●) and 55 patients with CC genotype (■). By general linear models repeated measure analysis for repeated measure data, rs6897932 was determined to exert no significant effects on the recovery of CD31^+^ RTEs counts and sjTREC levels after the cessation of chemotherapy (*p* > 0.05). Data are shown as means ± SD.

**Table S1. Baseline characteristics in patients with different genotypes of rs7718919 and rs6897932**

| **Characteristics** | **rs7718919** | | ***P*** | **rs6897932** | | ***P*** |
| --- | --- | --- | --- | --- | --- | --- |
|  | **GT + TT (n = 21)** | **GG (n = 63)** |  | **CT + TT (n = 29)** | **CC (n = 55)** |  |
| Age (years), median (IOR) | 37 (18–67) | 42 (18–67) | 0.218 | 38 (19–66) | 42 (18–67) | 0.376 |
| Sex, m/f | 9/12 | 30/33 | 0.705 | 14/15 | 25/30 | 0.805 |
| CD4^+^ T cells (×10^9^/L) | 586.72 ± 383.86 | 618.70 ± 384.23 | 0.603 | 613.68 ± 368.19 | 549.62 ± 320.76 | 0.487 |
| RTE (×10^9^/L) | 154.84 ± 132.07 | 120.42 ± 123.91 | 0.219 | 153.65 ± 132.31 | 138.49 ± 78.35 | 0.326 |
| TREC (copies/10^6^ PBMCs) | 6771.36 ± 7515.30 | 5810.91 ± 6016.48 | 0.614 | 7671.57 ± 7308.09 | 5083.28 ± 2721.77 | 0.166 |

RTE: recent T cell emigrant; sjTREC: single-joint T-cell receptor rearrangement excision circle
